# Responses of Herbivorous Fishes and Benthos to 6 Years of Protection at the Kahekili Herbivore Fisheries Management Area, Maui

**DOI:** 10.1371/journal.pone.0159100

**Published:** 2016-07-27

**Authors:** Ivor D. Williams, Darla J. White, Russell T. Sparks, Kevin C. Lino, Jill P. Zamzow, Emily L. A. Kelly, Hailey L. Ramey

**Affiliations:** 1 Coral Reef Ecosystem Program, Pacific Islands Fisheries Science Center, National Oceanic and Atmospheric Administration, 1845 Wasp Boulevard, Building 176, Honolulu, Hawaii, United States of America; 2 Department of Land and Natural Resources, Division of Aquatic Resources, Maui Office, 130 Mahalani Street, Wailuku, Hawaii, United States of America; 3 Joint Institute for Marine and Atmospheric Research, 1000 Pope Road, Honolulu, Hawaii, United States of America; 4 Center for Marine Biodiversity and Conservation, Scripps Institution of Oceanography, University of California San Diego, 9500 Gilman Drive, La Jolla, California, United States of America; 5 225 Punakea Loop, Lahaina, Hawaii, United States of America; Leibniz Center for Tropical Marine Ecology, GERMANY

## Abstract

In response to concerns about declining coral cover and recurring macroalgal blooms, in 2009 the State of Hawaii established the Kahekili Herbivore Fisheries Management Area (KHFMA). Within the KHFMA, herbivorous fishes and sea urchins are protected, but other fishing is allowed. As part of a multi-agency monitoring effort, we conducted surveys at KHFMA and comparison sites around Maui starting 19 months before closure, and over the six years since implementation of herbivore protection. Mean parrotfish and surgeonfish biomass both increased within the KHFMA (by 139% [95%QR (quantile range): 98–181%] and 28% [95%QR: 3–52%] respectively). Most of those gains were of small-to-medium sized species, whereas large-bodied species have not recovered, likely due to low levels of poaching on what are preferred fishery targets in Hawaii. Nevertheless, coincident with greater biomass of herbivores within the KHFMA, cover of crustose coralline algae (CCA) has increased from ~2% before closure to ~ 15% in 2015, and macroalgal cover has remained low throughout the monitoring period. Strong evidence that changes in the KHFMA were a consequence of herbivore management are that (i) there were no changes in biomass of unprotected fish families within the KHFMA; and that (ii) there were no similar changes in parrotfish or CCA at comparison sites around Maui. It is not yet clear how effective herbivore protection might eventually be for the KHFMA’s ultimate goal of coral recovery. Coral cover declined over the first few years of surveys–from 39.6% (SE 1.4%) in 2008, to 32.9% (SE 0.8%) in 2012, with almost all of that loss occurring by 2010 (1 year after closure), i.e. before meaningful herbivore recovery had occurred. Coral cover subsequently stabilized and may have slightly increased from 2012 through early 2015. However, a region-wide bleaching event in 2015 had already led to some coral mortality by the time surveys were conducted in late 2015, at which time cover had dropped back to levels recorded in the KHFMA in 2012.

## Introduction

Concern about coral reefs’ vulnerability to a range of local and global threats has provoked considerable interest in management strategies that could help to check or reverse coral declines. Highlighting the role of coral reef herbivores in promoting conditions that favor corals, several researchers have proposed that protecting herbivorous fishes could help coral reefs to resist and recover from a variety of stressors [1–5]. Certainly, there is abundant experimental and correlative evidence that algal forms which tend to dominate in low herbivory conditions, macroalgae (i.e. fleshy, upright and/or structurally complex algae) and thick turfs, generally inhibit coral recruitment and growth, and can cause mortality when they come into contact with coral tissue; whereas algae that dominate in heavily grazed environments, i.e. crustose coralline algae (CCA) and sparse turfs, tend to facilitate coral recruitment and are benign or inferior competitors [6–17].

Thus it might be expected that there would be many examples of marine reserves in which restrictions on fishing of herbivores had led to clear positive outcomes for corals. Although there are examples of reserves that appear to have been effective in that regard [18–20], there are several others cases of long-standing marine reserves in which there is no evidence of effects of that kind [21–24]. Among the reasons why some reserves have not been effective in this regard are: (i) that herbivores did not recover within the reserve, either because they were not heavily fished prior to closure, or because of insufficient compliance [21,24,25]; (ii) that although herbivorous fishes recovered within reserves, any net positive effect was counteracted by a decline in herbivory by sea urchins, as urchin predators also recovered in the closed area [23]; and (iii) that although herbivores were initially depleted in reserves, problem algae had never become abundant [22]. Thus, prerequisites for herbivore protection within a marine reserve to be effective at restoring or sustaining coral cover probably include: (i) a reasonable expectation that herbivory will increase within the reserve after closure; and (ii) evidence that, absent protection, problem algae have been or would become sufficiently abundant to reduce coral viability. Furthermore, in locations where sea urchin predators are also targeted, limiting reserve protection to herbivores alone might be not only more palatable to local stakeholders than more comprehensive closure [5], but also more likely to lead to net increases in total herbivory.

Here we describe the effects of herbivore protection at a location that meets these criteria, the Kahekili Herbivore Fisheries Management Area (KHFMA) on Maui, Hawaii. The KHFMA, in which herbivorous fishes and urchins are protected but other forms of fishing are permitted, was created in response to concerns about condition of reefs in the Kahekili Beach/North Kaanapali area. Specific concerns include that there had been repeated ephemeral blooms of macroalgae from at least the late 1980s [26,27], and that coral cover at a nearshore survey site there had declined from ~55% in 1994 to ~35% in 2006 [28]. In spite of that decline in coral cover, at the time of establishment, reefs in this area were still in relatively good condition, largely consisting of structurally-complex and coral-dominated habitats.

Our strong expectation from multiple other marine reserve studies in Hawaii and beyond [29,30] was that herbivore biomass would likely increase within the KHFMA. However, it was less clear whether herbivory would increase sufficiently for benthic algal assemblages to shift to states more suitable for coral recovery–e.g. lower macroalgae and higher CCA cover, and how quickly that might ultimately lead to coral recovery. Using data from a consistent monitoring program beginning 19 months prior to closure and continuing for six years after establishment, we report on trends in fish, benthic, and urchin assemblages within the KHFMA and compare patterns of change with those at several comparison sites around Maui.

## Methods

### Study Area and Establishment of Herbivore Fisheries Management Area

**Fig 1:**
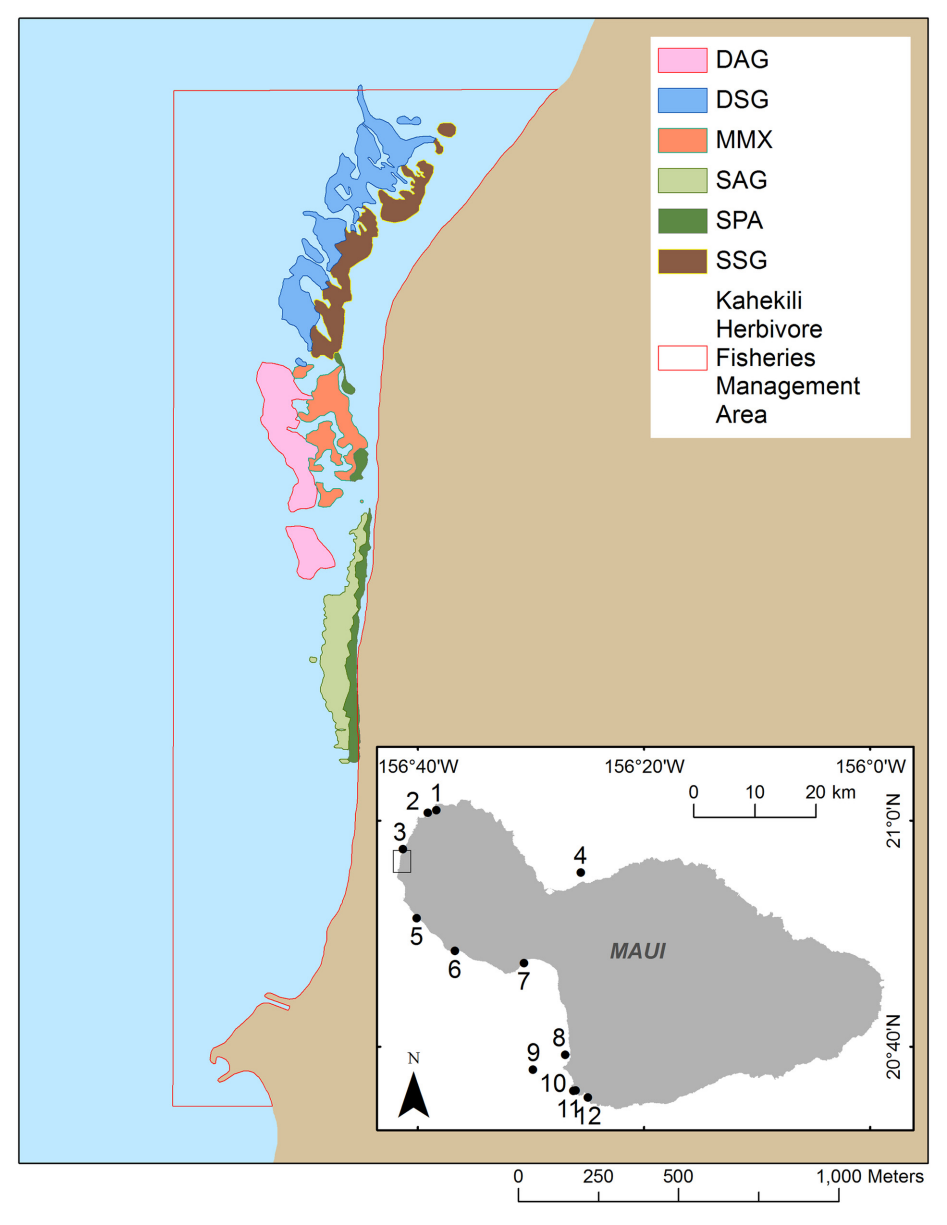
Map of Kahekili HFMA, with reef area classified into 6 habitat-strata. Strata: Deep Aggregate Reef (DAG); Deep Spur and Groove (DSG); Mixed Mid-Depth (MMX); Shallow Aggregate Reef (SAG); and Shallow Spur and Groove (SSG). Further detail on habitat types is given in the Methods section. Numbers 1–12 in the inset of Maui Island represent the location of monitoring comparison sites, where fish (F) and benthic (B) data are available from 2008 to 2015. 1 = Honolua MLCD (F+B); 2 = Kapalua Bay (F); 3 = Mahinahina (B); 4 = Papaula Point (B); 5 = Puamana (B); 6 = Olowalu (B); 7 = Maalaea (B); 8 = Makena/Keawekapu (F); 9 = Molokini MLCD (F+B); 10 = Ahihi-Kinau Natural Areas Reserve System (NARS) (F); 11 = Kanahena Point (B); 12 = La Perouse (F).

The KHFMA, which was established on July 25^th^ 2009, encompasses ~ 3 km of coastline and nearshore coral reef and associated habitats in West Maui, Hawaii (Fig 1). Several large resorts are situated close to shore along the KHFMA coastline, and more generally the reefs and waters inside the KHFMA and adjacent beaches are readily accessible and heavily used for a variety of recreational and commercial purposes, including fishing, swimming, snorkeling, and other ocean recreation.

Within the KHFMA, the take of surgeonfishes (Acanthuridae), parrotfishes (Labridae, tribe: Scarinae), chub (Kyphosidae) and sea urchins (Echinoidea) is prohibited, but there are no area-specific restrictions on fishing for other fishes or invertebrates [31]. While sea urchins are harvested in some parts of the Hawaiian Islands, we are not aware of there being a significant fishery for them on KHFMA reefs prior to closure, however they were included in the regulations as a precaution to ensure that one did not develop.

It is difficult to know what factors contributed to macroalgal blooms and apparent coral decline at Kahekili. Prior to establishment of the KHFMA, herbivorous fishes appear to have been depleted, but elevated groundwater nutrients impinging on the reef though multiple underwater seeps may also have played a role in the algal blooms [27]. Sources of excess nutrients in groundwater include the Lahaina Wastewater Treatment Plant, which discharges treated effluent into injection wells [32,33], as well as resort and golf course landscaping, and upslope agriculture—as large areas of West Maui were previously given over to sugar and pineapple plantations. Intensive sugar cultivation ended in 1999 and pineapple farming declined over time until being phased out in West Maui in 2008, with lands converted to lower intensity diversified crops or left fallow [34–36]. Assessing the likely impacts of land-use change is complicated by the fact that groundwater travel times on Maui are slow, and hence that residual effects of former cultivation on groundwater are possible for several years [34].

### Hawaii Coral Bleaching Events 2014 and 2015

At the time of our surveys in September 2015, the majority of corals within the KHFMA had bleached, including nearly all corals of some genera, and there were already signs of some mortality (pers. obs. IDW). The bleaching event within KHFMA was part of a region-wide event, which severely impacted corals across much of Maui and Hawaii Islands and beyond (pers. obs. IDW). There was a much less prolonged and widespread bleaching event in the summer of 2014, but we did not witness any bleached corals inside the KHFMA at the time of our surveys or during other visits that year.

### Survey Methods

Within the KHFMA there are several distinct reef types that broadly vary in relation to depth gradients and position along the shoreline. Therefore, we classified hard-bottom reef habitat within the KHFMA into 6 habitat-strata, corresponding largely with dominant benthic communities, physical structure, and depth (Table 1, Fig 1). Shallow reef areas are present very close to shore, and the deep edge of the reef we sampled was around 16 m deep. Beyond that depth, habitats appear to generally consist of sand and *Halimeda* beds [37]. The southern portion of the KHFMA consists of a sand habitat stretching for several hundred meters, and the northern edge of the KHFMA is dominated by a similar expanse of relatively flat pavement habitat with loose sediment and occasional patches of higher coral cover. Therefore, the area surveyed constitutes the great majority of shallow coral reef habitat within the KHFMA.

**Table 1:** Habitat-strata classification and proportional area.

Category	Description	Area (Ha) [%]
Shallow Pavement (SPA)	Largely flat, low-relief and low coral cover areas dominated by limestone pavement and loose sediment, typically ~2–5 m deep.	2.35 [8.5%]
Shallow Aggregate Reef (SAG)	Moderately or highly complex reef adjacent to shore, with moderate to high coral cover and considerable structure arising from coral growths ~2–8 m deep.	3.96 [14.3%]
Deep Aggregate Reef (DAG)	Same as SAG, but these are offshore areas of reef, generally ~7–16 m deep.	5.33 [19.3%]
Mixed Mid-depth (MMX)	Benthos generally dominated by loose sediment and sand with sparse corals, but with patches of higher coral cover, typically 3–8 m deep.	3.51 [12.7%)
Shallow Spur-and-Groove (SSG)	Shallow portion of spur-and-groove habitat, where spurs are distinct but less well developed than deeper areas (i.e. spur height generally <2.5 m); depths generally ~3–5 m.	4.96 [18.0%]
Deep Spur-and-groove (DSG)	Very well developed spur-and-groove habitat, with spur heights often 3–5 m or more in depths of ~4–15 m.	7.50 [27.2%]

Surveys were conducted in 1 or 2 ‘rounds’ per year, with rounds generally constituting a 3- or 4-day period of intensive surveys (S1 Table). Within each round we surveyed between 62 and 106 haphazardly located 25 m transects, and subsequently assigned those transects to different habitat strata based on their locations extracted from the GPS towed by each dive team. Each transect was surveyed by a pair of divers. The lead diver recorded the number, species, and size in 5 cm bins of fishes in two passes along the transect: fishes > 15 cm TL were recorded on a 4 m-wide outward belt, and fishes < 15 cm TL on a 2 m-wide belt during the return swim. The fish diver laid out a gray dacron transect line on the outward swim. The second diver followed behind the fish diver, conducting a photo-transect survey on the outward swim and recording the number and species of urchins in a 1 m-wide belt centered on the transect line during the return swim. For all benthic surveys, photographs were taken at 1 m intervals along the transect line. Prior to 2010, benthic photographs were framed within a plastic quadrat laid onto the transect line. Since 2010, photos were taken directly above the substratum with a 1 m-spacer pole used to maintain the camera at a consistent height above the benthos.

Benthic cover was extracted from 25 photos per transect using image analysis software. Following a commonly applied local standard [38], we analyzed 50 points per frame in the first year of sampling, but subsequently dropped that to 15 points per frame after an informal cost-benefit analysis of the initial data. To the extent possible hard corals were identified to species level and macroalgae to genera, and other benthic components to functional group, e.g. turf, sand, crustose coralline algae [CCA].

One observer (IW) gathered around half the fish survey data in each survey round, other than the September 2009 round, and since 2010 the majority of other fish survey data was gathered by two observers (KL and JZ). However, there was considerable turnover in the divers conducting the benthic and urchin component of the surveys. As we are not confident that all observers reliably identified all urchins to species, we pooled those recorded as *Diadema paucispinum* or *Echinothrix* into a single grouping. Similarly, we do not use data on *Echinometra* here, as there appeared to be high variability among observers in their counts of these patchily distributed, generally small and at least partially cryptic species. In any case, *Echinometra* were most conspicuously abundant in shallow pavement habitats where the majority of substrate was scoured pavement covered by turf algae, i.e. habitats where there was relatively little scope for changes in benthic cover following establishment of the KHFMA.

### Comparative Data from other long-term monitoring programs

We compared fish and benthic trends within the KHFMA with those at 12 long-term monitoring sites surveyed by Hawaii Division of Aquatic Resources (DAR) which did not change management status and for which data covering the 2008 to 2015 period were also available. Locations of those comparison sites, comprising 4 sites with fish data, 6 with benthic data and 2 with both fish and benthic data, are shown in Fig 1.

Benthic comparative sites were established by the Hawaii Coral Reef Assessment and Monitoring Program (CRAMP), but have been surveyed annually by DAR staff since before the onset of this study. Details of the CRAMP program and survey methods are available elsewhere [38,39]. In brief, each CRAMP site has two stations, typically in ~3 and ~10 m depth, with each station having 10 fixed 10 m transects allocated around a 100 m-long spine. CRAMP sites were photo-surveyed, with one photograph taken each meter, and those images were subsequently analyzed using methods and benthic categories that were extremely similar to those used for KHFMA image analysis.

Fish comparative sites are all long-term fish monitoring sites surveyed by Hawaii DAR since at least 2008, which contained both shallow (~3 m) and deep (~8–12 m) stations. Each of those sites was surveyed by means of timed swims starting from 5 fixed starting points (3 shallow stations, 2 deep stations). Two pairs of divers conducted each survey, both pairs beginning at the same starting point but swimming in opposite directions along the depth contour. Each pair of divers swam in parallel, with each diver recording the number, size and species of fishes 15 cm TL and larger in 5 m-wide belts (i.e. total belt width of 10 m per pair of divers). Total distance covered by the two dive-pairs was determined using a tracking GPS towed by divers, and varied between ~100 and ~450 m. The goal of these surveys is to assess populations of large-bodied targeted taxa, and divers do not record observations of non-targeted families or of two species of abundant small surgeonfishes (*Acanthurus nigrofuscus* and *Zebrasoma flavescens*).

### Data Synthesis and Analysis

Fish observation data was converted into biomass using length-to-weight conversion parameters originally taken from FishBase [40] or the Hawaii Co-operative Fishery Research Unit at the University of Hawaii. Transect-level reef fish biomass, urchin densities, and benthic cover were pooled into KHFMA-scale values by first calculating mean and variance within habitat-strata, and then calculating weighted KHFMA-scale mean and variance using the formulas given in [41], with habitat-strata weighted by their respective sizes (Table 1). Data per round were pooled to annual and multi-year values using the same formulas, weighting individual rounds and years equally. At comparison benthic sites, the 2 stations surveyed were weighted equally to generate annual mean and variance per site. At comparison fish sites, the 2 depth strata (‘shallow‘ and ‘deep’) were weighted equally.

To assess change in the KHFMA or at comparative locations around Maui over the same time period, we used bootstrapping with 10,000 iterations and calculated with a correction for small sample size [42] to generate 95% quantile range (95% QR) of differences in fish biomass or benthic cover between the 2 years leading up to establishment of the KHFMA (‘before’: 2008–9) and the most recent 2-year period (‘after’: 2014–15). 95% QR of difference not overlapping zero is taken as evidence of significant difference at alpha = 0.05, and displaying results in this way (i.e. as mean and quantile range of difference) provides substantial additional information compared to simply reporting a significance test result [43].

We used 2-year before-and-after periods because (i) we had 2 years of pre-closure data from KHFMA (the September 2009 sampling round was conducted 4–6 weeks after establishment, but reserve boundary and rule signs were not posted until November 2009); and (ii) we wanted to use consistent before and after periods for KHFMA and Maui comparative sites, and there was not sufficient data from Maui comparative fish sites to compare between single years. Specifically, there were between 2 and 5 fish surveys at several of the comparative sites in single years, but over any 2-year period, the lowest number of fish surveys at any comparative site was 12. Comparisons of before-after change at KHFMA and other Maui fish survey sites were made using consistent subsets of the fish assemblages. Specifically, for that analysis we excluded fishes not counted as part of the survey protocol implemented at the Maui comparative sites from the KHFMA count data, i.e., all fishes smaller than 15 cm TL and all observations of the two small surgeonfishes (*A*. *nigrofuscus*, *Z*. *flavescens*).

### Ethics Statement

No specific permissions were required at the study location, as we conducted only visual survey work and did not collect or otherwise damage any marine organisms. For the same reason, we did not require approval by an animal ethics committee for our survey methods.

## Results

### Fish Biomass inside KHFMA

**Fig 2:**
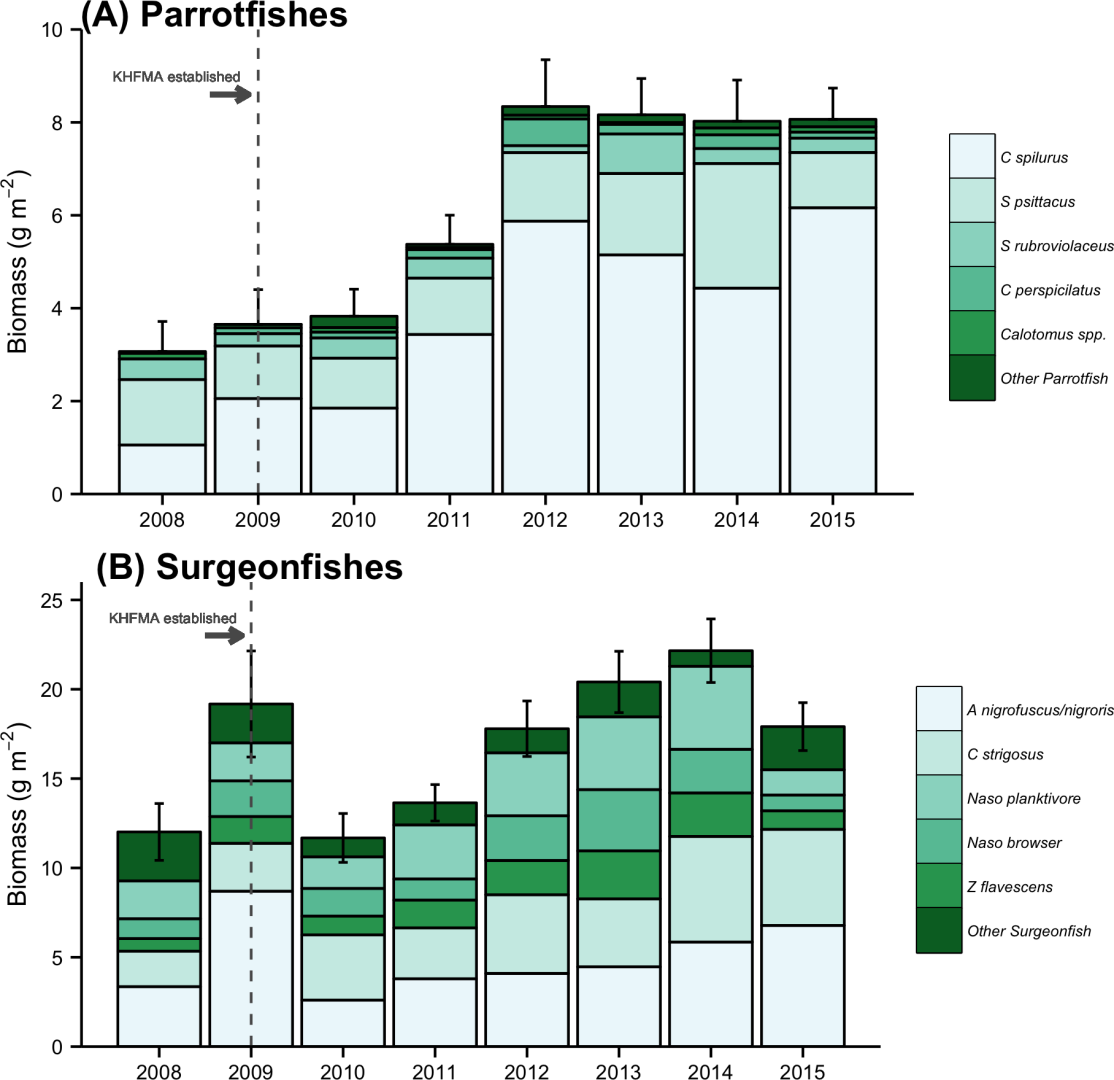
Trends in biomass of herbivorous fishes. Error bars represent standard error by family. ‘*Naso* browser’ are *N*. *uncornis* and *N*. *lituratus*, and ‘*Naso* planktivore’ are made up of species that primarily feed on plankton as adults, *N*. *hexacanthus* and *N*. *brevirostris*.

**Fig 3:**
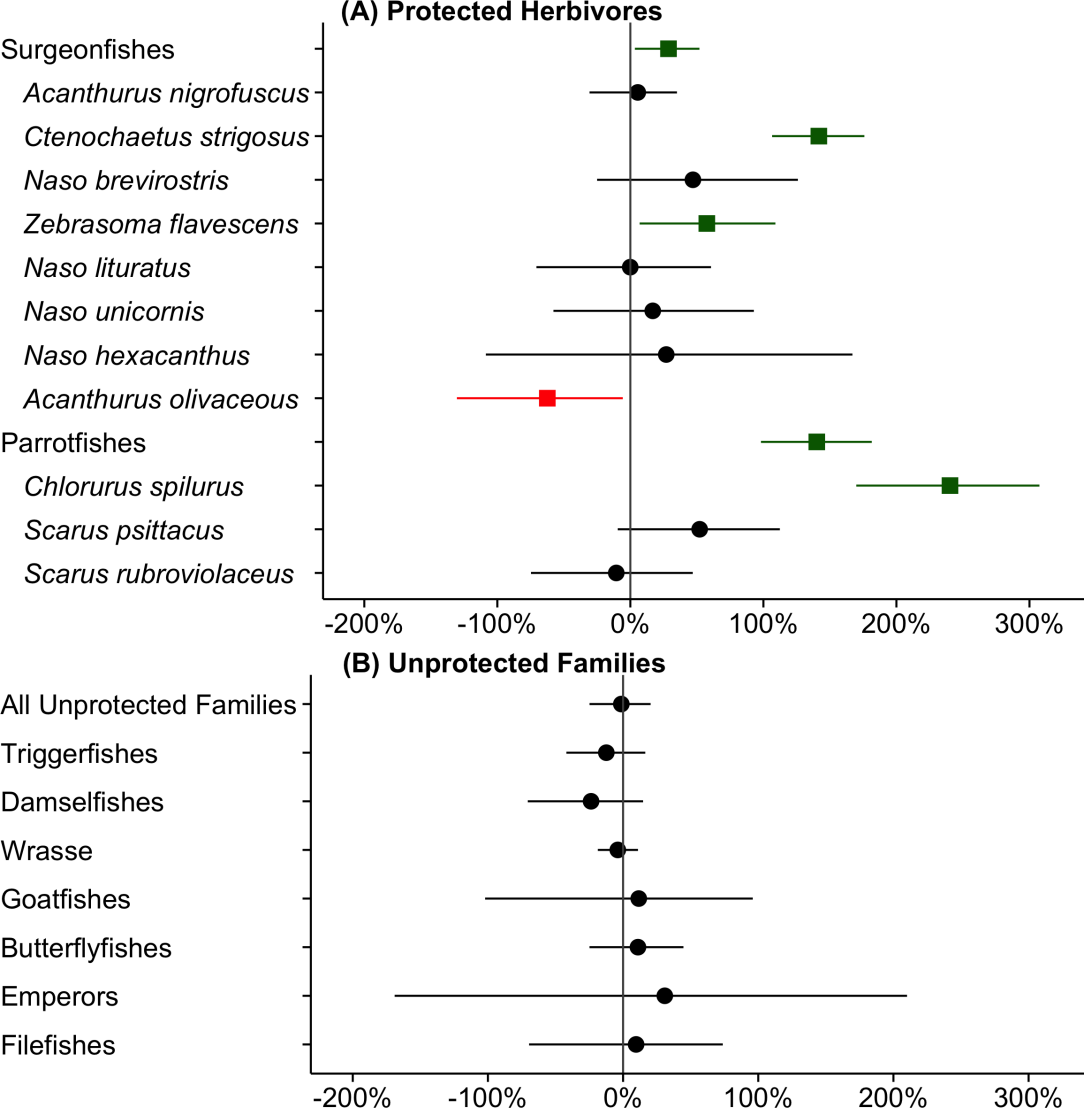
Net change between ‘before’ (2008–9) and ‘after’ (2014–15) by taxa for herbivores (protected from fishing) and other families (without fishery restriction). Data points represents the net proportional change in biomass from ‘before’ to ‘after’, and lines present the 95% quantile range (95%QR) of that change. 95%QR not overlapping zero is evidence of a significant difference between time periods, shown as a green square (biomass increase) or red square (biomass decrease). Taxa shown are all those with mean biomass across before and after periods of at least 0.5 g/m^2^, plus a large bodied parrotfish species, *Scarus rubroviolaceus*, with mean biomass slightly below that level. Within groupings (surgeonfishes, parrotfishes, unprotected families), taxa are ordered by mean biomass from highest to lowest.

Parrotfish biomass increased rapidly after establishment of the KHFMA in 2009, approximately plateauing from 2012 onwards (Fig 2). Between 2008–9 and 2014–15 parrotfish biomass increased by 139% (95% QR: 98 to 181%, Figs 2 and 3). The majority of increase was of one species, *Chlorurus spilurus*, which increased by 241% (95% QR: 170 to 308%, Fig 3); mean biomass of another common parrotfish species, *Scarus psittacus*, increased by 52%, but that increase was not significant (95%QR: -10 to 112%, Fig 3). Biomass of all other species of parrotfishes made up only 12% of family biomass over the study period.

**Fig 4:**
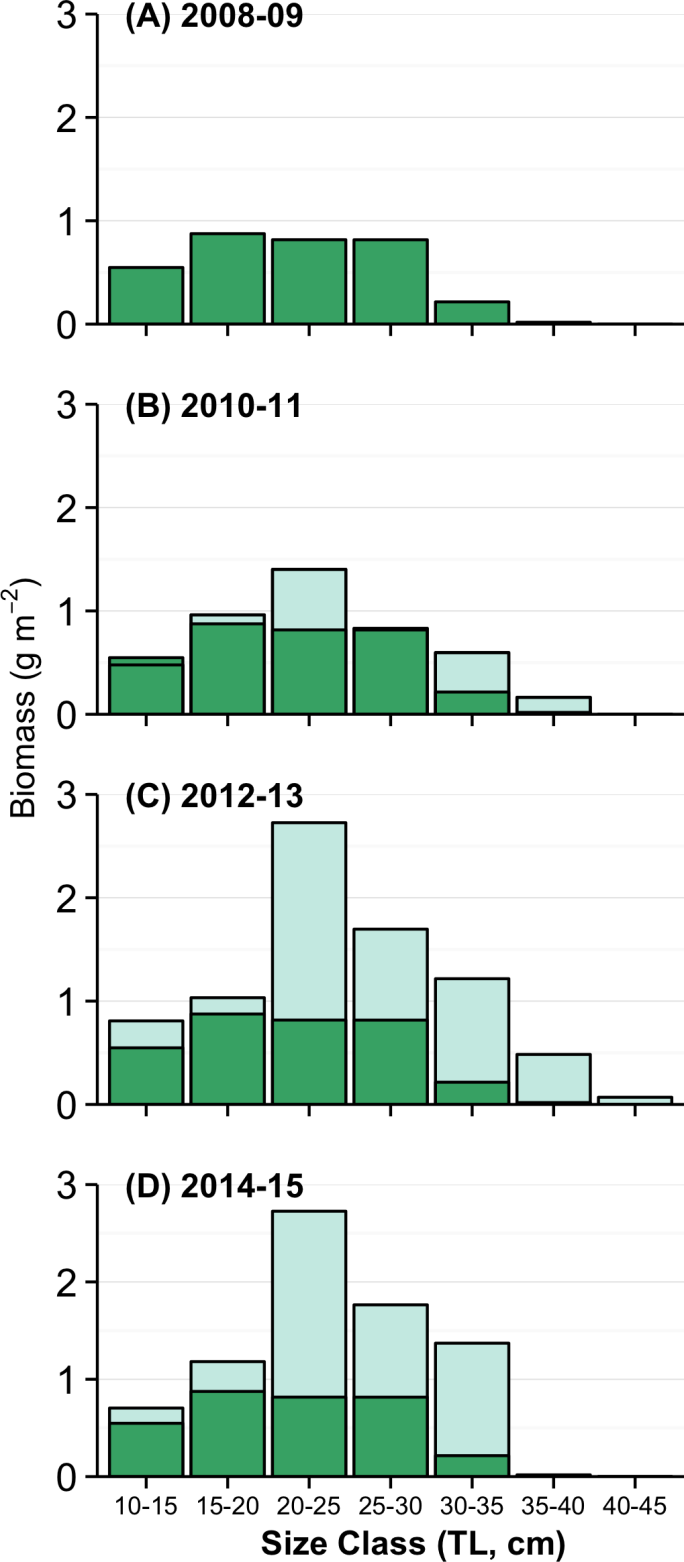
Size distribution of parrotfishes 10cm and above, pooled into 2-year periods. 2008–9 represents size distribution before closure. The dark green columns in all figures represent biomass per size class in 2008–9, and are shown in later figures to highlight differences between 2008–9 and later time periods.

The increase in parrotfish biomass was nearly all in size classes 20 cm and above, with biomass in larger size classes steadily increasing over the first few years of protection (Fig 4). Prior to protection, few parrotfishes larger than 30 cm were recorded during surveys, but by 2012 and in subsequent years, those relatively large fishes made up a substantial portion of parrotfish biomass (Fig 4).

Estimated biomass of surgeonfishes was anomalously high in 2009, mainly due to encounters with large roving schools of *Acanthurus nigrofuscus* in shallow habitats, but otherwise appeared to trend upwards after closure, until declining slightly in 2015 (Fig 2). In spite of the high counts in 2009, total surgeonfish biomass increased by 28% (95% QR: 3 to 52%) between 2008–9 and 2014–15 (Fig 3), with significant increases for two species, *Zebrasoma flavescens* and *Ctenochaetus strigosus*, which increased by 58% (95%QR: 7 to 109%) and 142% (95%QR: 107 to 176%) respectively (Fig 3). Over the same time period, biomass of one species of surgeonfish, *Acanthurus olivaceus*, declined by 62% (95% QR: -6% to -130%, Fig 3).

Notably, although there were significant or near significant biomass increases for several species of surgeonfish and parrotfish, there were no indications of an increase for several relatively large-bodied species of those families, including *N*. *uncornis*, *N*. *lituratus* and *Scarus rubroviolaceus* (Fig 3). Trends over time (Fig 2 and S1 Fig) together with our impressions from spending several days diving on the site at least twice a year was that those species were more frequently observed by around 2012/13, but their numbers subsequently declined so that by end of 2015 there was essentially no difference in their pre- and post-closure biomass (Fig 3 and S1 Fig).

Although they are present around near-shore rocky and boulder habitats at the southern edge of the KHFMA, no chub were recorded during surveys inside the KHFMA, indicating that they were rare to absent in the sampled habitats.

In contrast to the patterns for herbivorous families, there was no significant or near significant increase in biomass of 9 non-target families considered—those being all non-target families with mean biomass > 0.5 g m^-2^ (Fig 3).

### Changes in herbivorous fishes at KHFMA and Maui Comparison Sites

**Fig 5:**
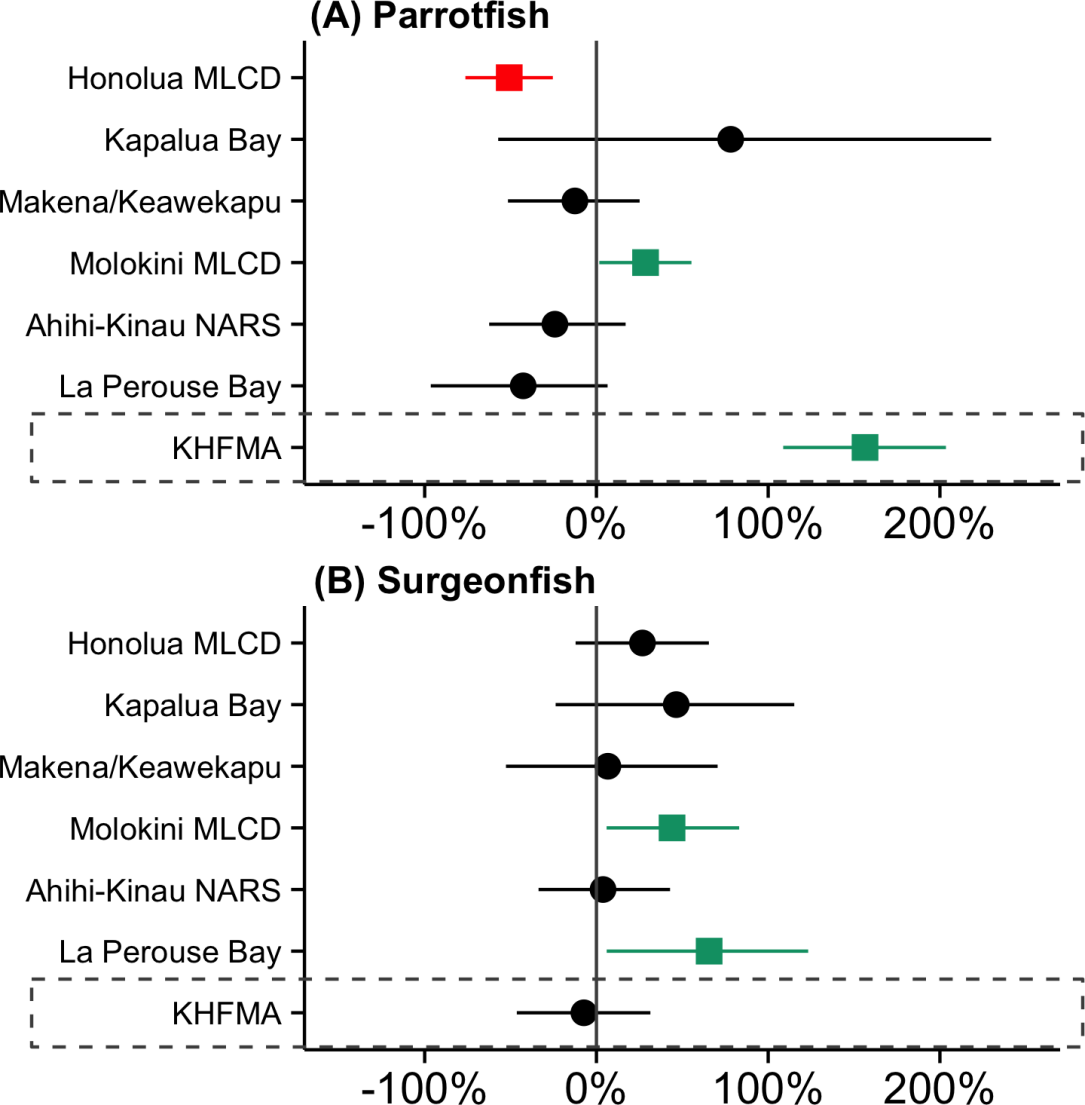
**Proportional change between ‘before’ (2008–9) and ‘after’ (2014–15) in biomass of (a) parrotfishes >15 cm and (b) surgeonfishes > 15 cm at KHFMA and comparison locations surveyed by Hawaii DAR.** Data points represent mean change in biomass, and lines are 95% quantile range (95%QR) of that change. 95%QR not overlapping zero is evidence of a significant difference between time periods, shown as a green square (increase) or red square (decrease). As two species of surgeonfish (*A*. *nigrofuscus* and *Z*. *flavescens*) and all fishes < 15 cm TL are not recorded during surveys at Maui comparison sites, observations of those species or size class are also not included in KHFMA data for this figure.

The large increase in biomass of parrotfishes 15 cm TL and above at KHFMA between 2008–9 and 2014–15 (+157%, 95% QR: 109–240%, Fig 5) was not matched at any of the 6 comparison sites. Among those sites, mean biomass declined at 4 and increased at 2, but the only significant changes were a decrease at Honolua Bay (-51%, 95% QR: -25% to -76), and a relatively small increase at Molokini (29%, 95% QR: 2 to 55%, Fig 5 and S2 Table). The only indication of a substantial increase in parrotfish biomass at a comparison site was at Kapalua Bay, but the increase there was far from significant (95% QR: -57% to 230%), and the biomass increase was very low in absolute terms, as even in 2014–15, mean biomass there was substantially lower than at any other surveyed site (S2 Table). Between 2008–9 and 2014–15 mean biomass of 15 cm and larger parrotfishes at KHFMA changed from being close to the average of the Maui comparison sites (2008–9 KHFMA: 2.7 g m^-2^; Maui comparison sites 2.6 g m^-2^) to being more than double the mean of the comparison sites (2014–15 KHFMA: 7.0 g m^-2^; Maui comparison sites 3.2 g m^-2^, S2 Table).

For the subset of surgeonfishes that were recorded during surveys at Maui comparison sites (15 cm TL and above, and excluding *A*. *nigrofuscus* or *Z*. *flavescens*), there was no significant change inside at KHFMA between 2008–9 and 2014–15 (Fig 5 and S2 Table). In contrast those increased at 2 of the 6 Maui comparison sites (Molokini MLCD and La Perouse Bay, Fig 5 and S2 Table).

### Sea Urchins

Estimated sea urchin densities varied considerably between rounds, but there was no indication of a significant trend in those relating to establishment of the KHFMA (S2 Fig). However, the density of *Tripneustes gratilla* was 25% higher in 2014–15 than in 2008–9 (95% QR: 1 to 49%).

### Benthic Cover

**Fig 6:**
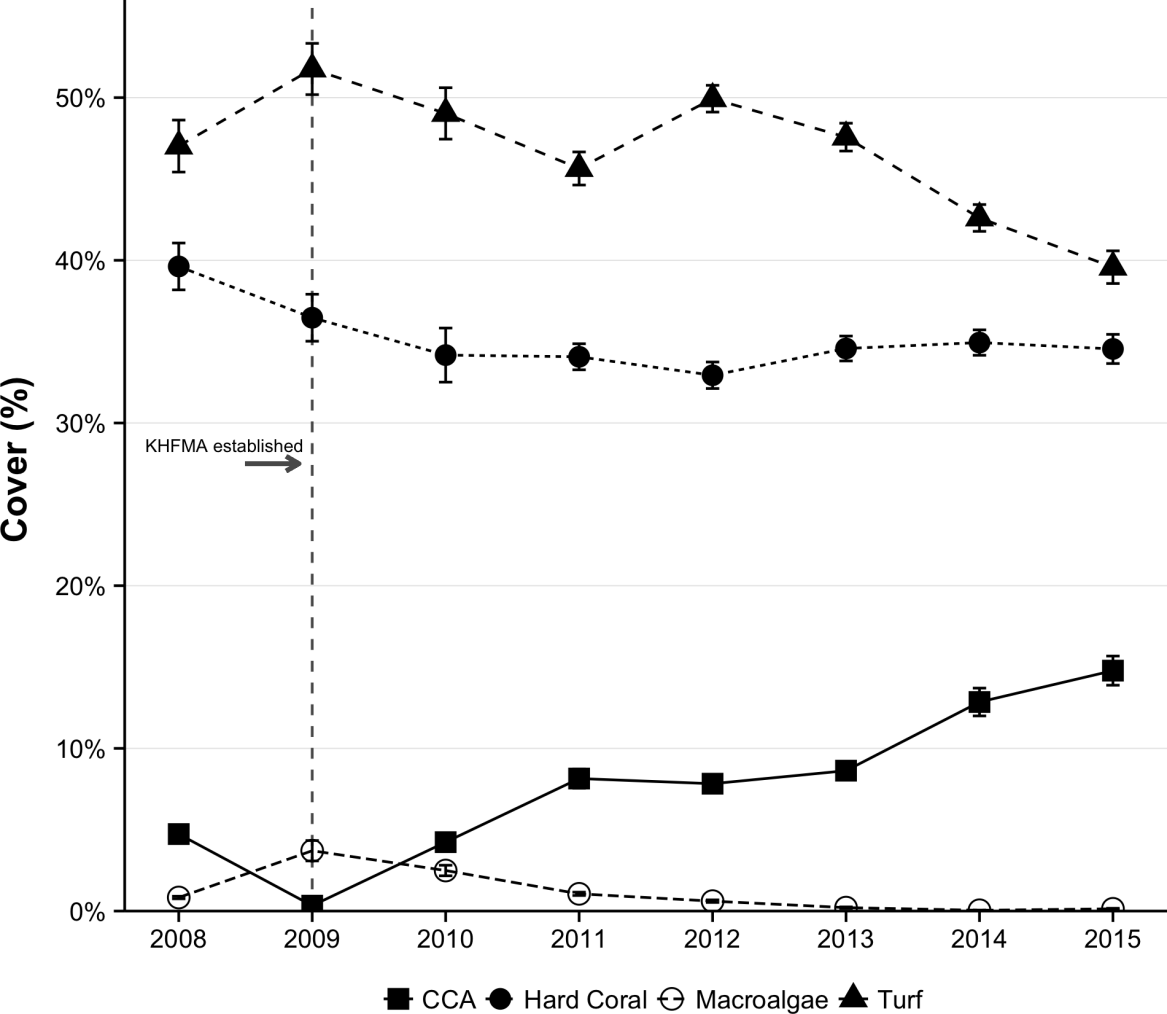
Trends in benthic cover at KHFMA. Data shown are annual mean and standard error.

Coral cover declined over the first few years of monitoring, most particularly between 2008 and 2010, reaching a low in 2012 (Fig 6), and changing from 39.6% ± 1.4% (mean ± SE) to 32.9% ± 0.8% over that period − a net loss of 17% of the previous cover (95% QR: -9% to -25%). Coral cover subsequently stabilized, and appeared to trend upwards, to 34.9% ± 0.8% in 2014 (Fig 6). Coral cover was marginally higher in the first round of surveys in 2015, but by the time of the second survey round, the 2015 bleaching event was well underway, many corals in the KHFMA were bleached and there appeared to already be some associated mortality, with the net effect that mean cover for all of 2015 was slightly lower than in 2014 (34.6% ± 0.9%). The great majority of coral was of 2 genera: *Porites*, which made up 75% of cover over the entire time period, and *Montipora* (22% of all cover).

Macroalgae cover was low at the time of all survey rounds, but has been virtually nil in most recent years, with highest cover in any year being 3.7% ± 0.6% in 2009 (Fig 6). The clearest change in benthic communities since establishment of the KHFMA has been a steady and substantial rise in crustose coralline algae (CCA), which increased from 2.5% ± 0.3% in 2008–9 to 13.8% ± 0.6% in 2014–15 (Fig 6). Turf algae was the largest component of the benthic assemblage in all years, ranging between 51.8 ± 1.6% in 2009 to 39.6 ± 1.0% in 2015.

**Fig 7:**
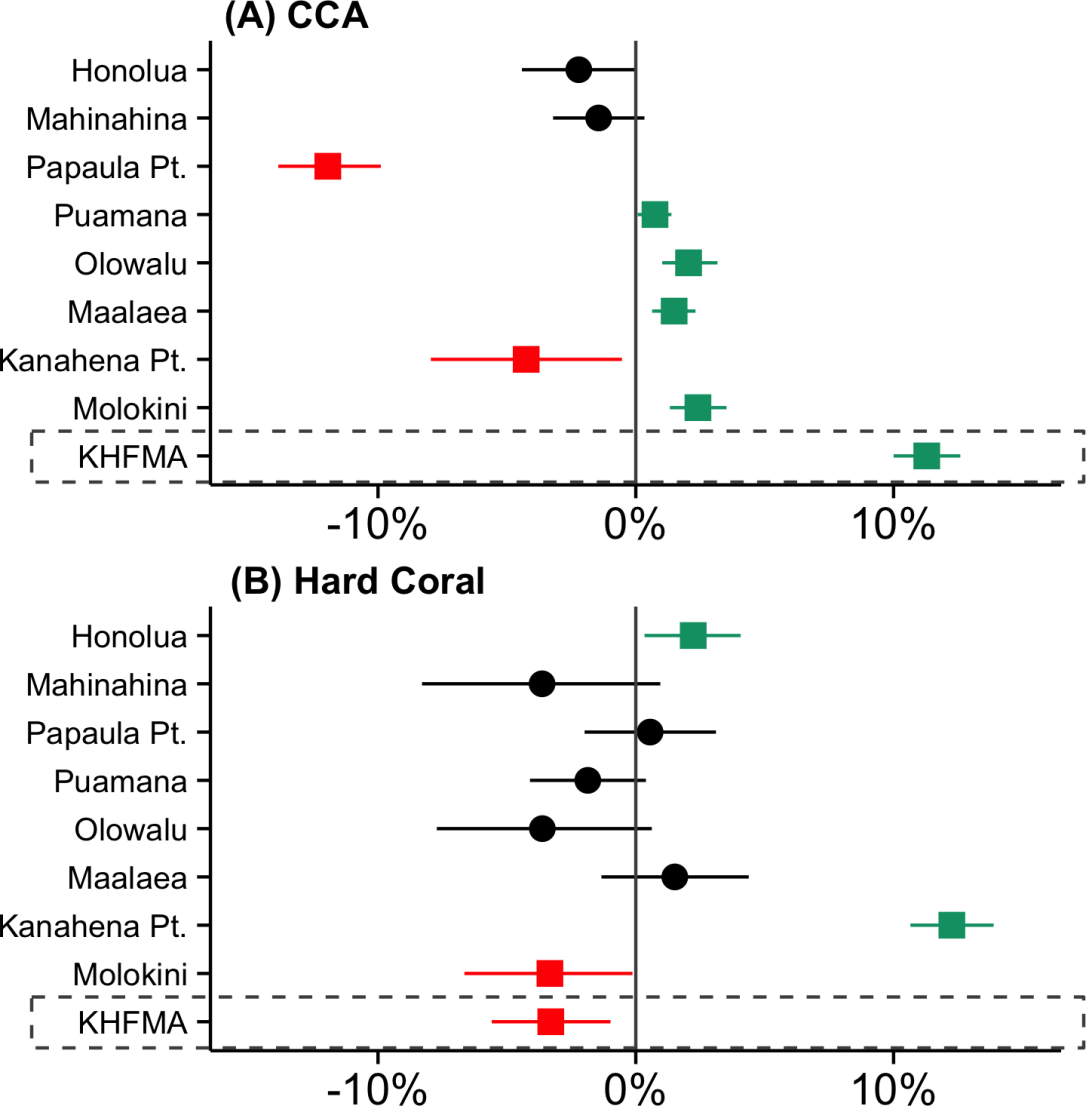
**Net change between ‘before’ (2008–9) and ‘after’ (2014–15) for cover of (a) crustose coralline algae (CCA) and (b) hard coral at KHFMA and comparison locations surveyed by Hawaii DAR.** Data points represent the net absolute change in % cover from ‘before’ to ‘after’, and lines are 95% quantile range (95%QR) of that change. 95% QR not overlapping zero are taken as evidence of a significant difference between time periods, shown as a green square (increase) or red square (decrease).

The large increase in CCA at KHFMA was not evident at the 8 Maui comparison sites from which we have data (Fig 7). CCA cover increased at 4 of those sites, and declined at 2, but the magnitude of change at sites showing an increase was ~1–2% (S3 Table), far less than the > 10% rise in CCA cover at KHFMA (S3 Table). There was also no clear trend in coral cover across those comparison sites between 2008–9 and 2014–15, with cover declining at 1 site and increasing at 2 of those sites (Fig 7). The notably large increase in coral cover at one of those sites, Kanahena Point (Fig 7), represents recovery from mortality caused by a crown of thorns outbreak in 2006 (pers. obs. RTS).

## Discussion

Reef fishes generally take many years, or decades, to reach post-closure maxima within reserves, and surgeonfishes, which include many long-lived species [44,45], are generally among the slowest to recover [23,46,47]. Potential secondary effects, such as coral recovery, will inevitably lag behind changes in fish populations [48,49]. Thus, although we have six years of post-closure data, it will take considerably more time before we can fully assess the effectiveness of herbivore management on benthic communities in the KHFMA. However, there have already been substantial changes consistent with the goals of the intervention inside the KHFMA including increased biomass of herbivorous fishes, particularly of parrotfishes, and in what we assume to be the consequences of increased herbivory, namely a five-fold increase in CCA cover.

Macroalgae were never abundant in the KHFMA during the course of our study–maximum cover was 4% in 2009, and has subsequently declined to be ~ 0.1% in recent years. Also, there were regular summertime macroalgal blooms including in 2001 [27], 2005, and 2006 (pers. obs.), but there have been no macroalgal blooms that we are aware of since 2006. The dominant algae throughout our study − turf algae − constitute a very wide range of algal communities from sparse heavily cropped turfs through to thick mixed turf assemblages, with likely very different effects on coral growth, recruitment and mortality [50,51]. In our experience it is difficult to classify benthos into different turf types from photo-transects, but with hindsight–given the lack of importance of macroalgae at the study location—it would have been desirable to have some capacity to distinguish among those turf types. Our clear impression from diving on KHFMA reefs over the last 8 years and more is that there has been substantial change in the benthos–not only in the increase in CCA, but that there are now many more patches of reef with conspicuously high cover of CCA (S4 Table) and heavily cropped turfs, i.e., substrate that appears nearly bare. Thus, we have reason to believe that herbivore management in the KHFMA has altered benthic algal assemblages more than might be evident from changes in CCA alone, and that conditions are probably substantially improved for coral growth and reproduction.

Evidence that the changes to algal assemblages had already or could eventually become sufficient to lead to meaningful reef-scale coral recovery is still somewhat inconclusive. KHFMA coral cover at the end of out study was actually lower than in 2008/9 (i.e. pre-closure). However, the net loss was due to a decline in coral cover occurring at the time of establishment of the KHFMA and continuing for the first few years after closure. That downward trend largely halted coincident with when herbivore biomass began to recover, and around the time when we started to see substantial increases in CCA, i.e. around 2010/11. Coral cover has been relatively stable since that time, but there are indications that it had begun to increase from its low in 2012 (32.9% ± SE 0.8%) through early 2015, at which time coral cover was 35.4% ± 1.3% (95%QR of net change in cover: -0.6% to +5.5%). Trends in coral cover within the KHFMA were therefore highly consistent with the results from a meta-analysis study of the effects of marine protected areas (MPAs) on coral cover, which showed an overall decline in coral cover in open areas and recently established MPAs, but that after around four years of closure and beyond MPAs had increasingly positive impacts on coral cover [49].

Unfortunately, reefs across much of Hawaii, including inside the KHFMA, experienced a mass-bleaching event in 2015. At the time we surveyed the reef in September 2015, the majority of corals had been bleached for some weeks and there appeared already to be some associated mortality. Thus it was not surprising that coral cover had declined (to 33.7% ± 1.2%). The 2015 bleaching event continued for around two months after our visit, and it seems likely we will see evidence of considerably more coral mortality once we are able to quantify the impacts of that event. Given the expectation of increased frequency and severity of bleaching events in coming years [52,53], there is a pressing need to better understand the extent to which local interventions such as herbivore protection may be able to provide some degree of mitigation [54,55]. Certainly, we expect this to become an increasingly important research focus for us at KHFMA and comparison sites.

A major driver for the creation of the KHFMA was to improve the local reef’s ability to deal with recurring summertime macroalgal blooms. During the time when the KHFMA was being established we had no reason to believe that macroalgal blooms would not be an ongoing concern. As it transpired, there have been no such blooms on Kahekili reefs since 2006, i.e. 3 years prior to closure, and overall macroalgae has become a negligible component of benthos across the KHFMA as whole. As we describe above, it seems highly likely that nutrients from agricultural sources have declined substantially over the last decade or more, which makes it difficult for us to distinguish between impacts of herbivore protection and changes in upslope land use. We have not been able to locate any definitive information on trends in the amount of nutrients entering Kahekili waters or at our other long-term sites, and thus are not able to incorporate that into our analysis. In addition, we have no data on the movement of fishes across KHFMA boundaries. Although many species are capable of large-scale movement on occasion, we think net migration is unlikely to be a major factor in the changes in herbivorous fishes assemblages that we observed, as: (i) the wide expanses of low-relief sand and pavement habitat to the north and south of the main KHFMA reef areas will provide some barrier to movement; and (ii) many, but not all, of the local herbivorous fish species typically have home ranges that are much smaller than the scale of the KHFMA [56–59]. Acknowledging those limitations, we note that changes to fish assemblages are highly consistent with those being the result of herbivore protection. First, the increase in parrotfish biomass occurred as a gradual accumulation of biomass in larger size classes which would be most affected by fishing, and which incidentally are likely to be particularly important grazers [7,60,61]. Secondly, there were no positive effects on unprotected (non-herbivorous) families. Furthermore, the decline in intensive sugar and pineapple cultivation was not restricted to the Kahekili watershed, and still there were not similar changes in parrotfish or CCA at the multiple comparative sites around Maui. Thus we believe it is likely that the dominant changes we have observed at KHFMA–increased biomass of parrotfishes and cover of CCA–were primarily a consequence of herbivore protection.

For this study, we chose not to match our KHFMA sampling with control sites that we could also track through time, and instead we took advantage of existing monitoring programs around Maui. We did that in part because of the lack of ideal control areas. Specifically, there was no other local reef we were aware of with the same diversity of reef types as the KHFMA − including aggregate reef, pavement, and extremely well developed spur and groove. Additionally, monitoring any number of control sites requires that survey effort to be split, potentially several times − if more than one control site is used. In this case, we were able to compare changes within the KHFMA with trends at 6 fish sites and 8 benthic sites spread widely across Maui. Although there were some disadvantages, including that we could only compare changes in fishes for a subset of the total assemblages (i.e. fishes > 15cm TL and excluding some common species), we believe this strategy gave us the ability to precisely quantify patterns of change inside the KHFMA, and to compare those with a wide range of Maui reefs. We suggest that this approach could be more generally suitable in situations where there are ongoing long-term monitoring programs willing to make data available for this purpose.

Compared to other reefs in the Pacific, Hawaii parrotfishes are relatively depauperate, with the great bulk of biomass on most reefs in the Hawaiian islands made up of 4 species–*Chlorurus spilurus*, *Scarus psittacus*, *C*. *perspicillatus* and *S*. *rubroviolaceus*. The two species, *C*. *spilurus* and *S*. *psittacus*, that comprised the bulk of parrotfish biomass and nearly all the gains within the KHFMA are relatively small-bodied and short-lived, with lifespans of 9 and 6 years respectively [62,63], and are probably therefore more able to quickly recover following closure than *S*. *rubroviolaceus* and *C*. *perspicillatus*, which have lifespans of > 20 years [64]. The apparent recent declines (post-2013) of those large-bodied parrotfishes, and of relatively large-bodied surgeonfishes, *Naso unicornis* and *N*. *lituratus*, strongly suggests that there has been some poaching of what are highly desirable fishery targets in Hawaii [57,65], as it seems unlikely that several such long-lived species would simultaneously decline after showing gradual but steady increases through the first years of closure (S1 Fig). As those large-bodied species comprise a large portion of two distinct herbivore functional groups on Hawaiian reefs–‘large excavators’ and ‘browsers’ [66]–and because their starting abundance was relatively low in the KHFMA, it seems likely that even relatively low levels of poaching are enough to maintain those species at very low levels and thus prevent the full range of herbivores from becoming established at KHFMA. Thus, it seems that both long-term and high-compliance closure will be necessary for complete recovery of herbivory inside the KHFMA, and likely at other potential herbivore management areas in Hawaii and beyond.

Overall, despite imperfect compliance, herbivore management at KHFMA has clearly been effective at increasing herbivore biomass and herbivory, and in turn that appears to have led to desirable changes to reef benthic assemblages, most notably a dramatic rise in cover of CCA. Given the short amount of time after closure of the KHFMA and before the 2015 bleaching event, the stabilization of coral cover was perhaps as positive an outcome as we could have reasonably expected. Because of the severity of that bleaching event and the reasonable likelihood of recurrence, it seems unrealistic to expect short-to-medium term recovery of coral cover to levels reported in the mid 1990s, e.g., 55% on nearshore reefs [28]. Instead, our near-term research focus at KHFMA and at comparison sites around Maui will be on extent of, and recovery from, bleaching-associated mortality. There is certainly evidence that reefs without abundant herbivores are more vulnerable to persisting loss of coral cover following disturbances such as major bleaching events and hurricanes [67–69]. Thus, the substantial increases in herbivore stocks and herbivory that have occurred within the KHFMA along with the large increases in CCA cover may be very important in increasing the resilience of those reefs to recent and future disturbances.

## Supporting Information

S1 Fig Trends in large- and small-bodied surgeonfishes and parrotfishes. Data shown are mean and standard error.
(DOCX)

S2 Fig Abundance of sea urchins at KHFMA. Data shown are annual mean and standard error.
(DOCX)

S1 Table Sampling Rounds, Survey Dates and # Transects. (DOCX)

S2 Table Mean and SE of parrotfish and surgeonfish biomass before closure (2008–9) and in 2 most recent year (2014–15) at KHFMA and at comparative locations around Maui. 95% quantile range (95%QR) not overlapping zero indicates significant change at alpha = 0.05, and are shown in bold. Kahekili HFMA data are shown for all fishes recorded during Kahekili surveys, and for the subset of fishes recorded at comparable sites (i.e. fishes > 15 cm TL, and excluding two common small-bodied surgeonfishes, *A*. *nigrofuscus* and *Z*. *flavescens*.
(DOCX)

S3 Table Mean and SE of cover of hard coral and crustose coralline algae (CCA) before closure (2008–9) and in 2 most recent year (2014–15) at KHFMA and at comparative locations around Maui. 95% quantile range (95%QR) not overlapping zero indicates significant change at alpha = 0.05, and are shown in bold.
(DOCX)

S4 Table Proportion of photoquadrats with >10%, >20%, >30% CCA over time. (DOCX)
