# Supplementary material for: Responses of Herbivorous Fishes and Benthos to 6 Years of Protection at the Kahekili Herbivore Fisheries Management Area, Maui
Source: PLoS One. 2016 Jul 27;11(7):e0159100. doi: 10.1371/journal.pone.0159100 (PMC4963024; doi:10.1371/journal.pone.0159100)
Supplement: S1 Fig — Data shown are mean and standard error. (DOCX) [file pone.0159100.s001.docx]

**S1 Fig. Trends in large- and small-bodied surgeonfishes and parrotfishes.** Data shown are mean and standard error

| 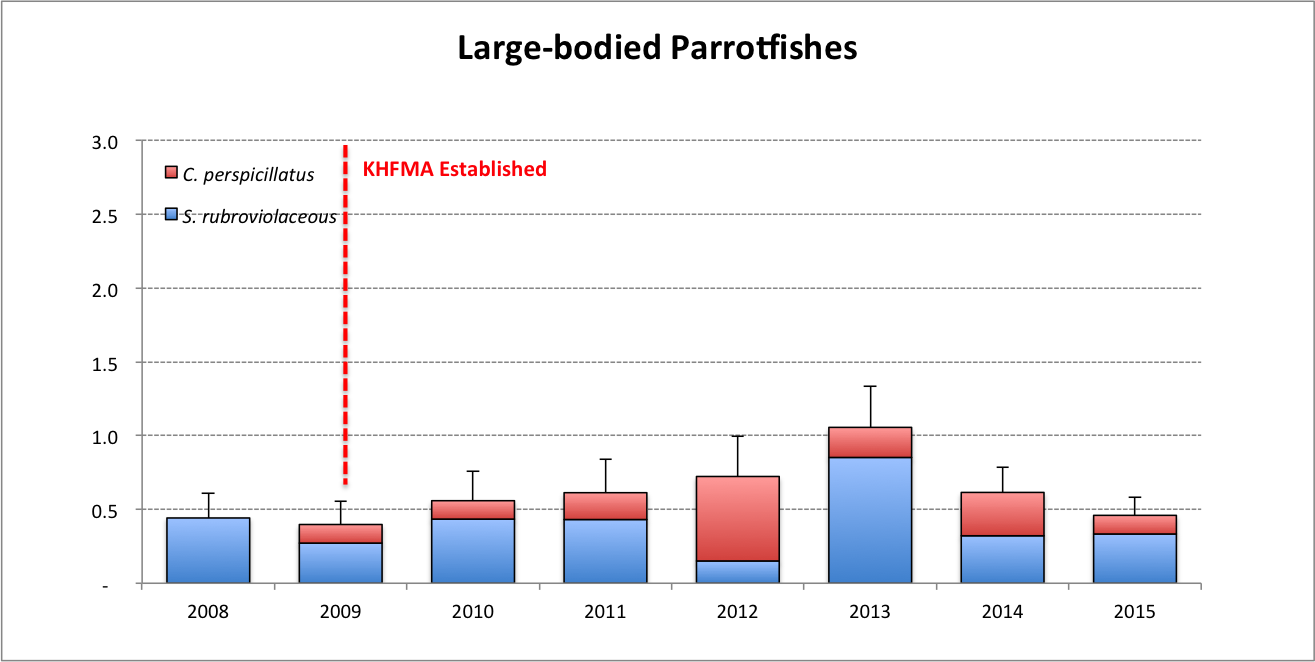 | 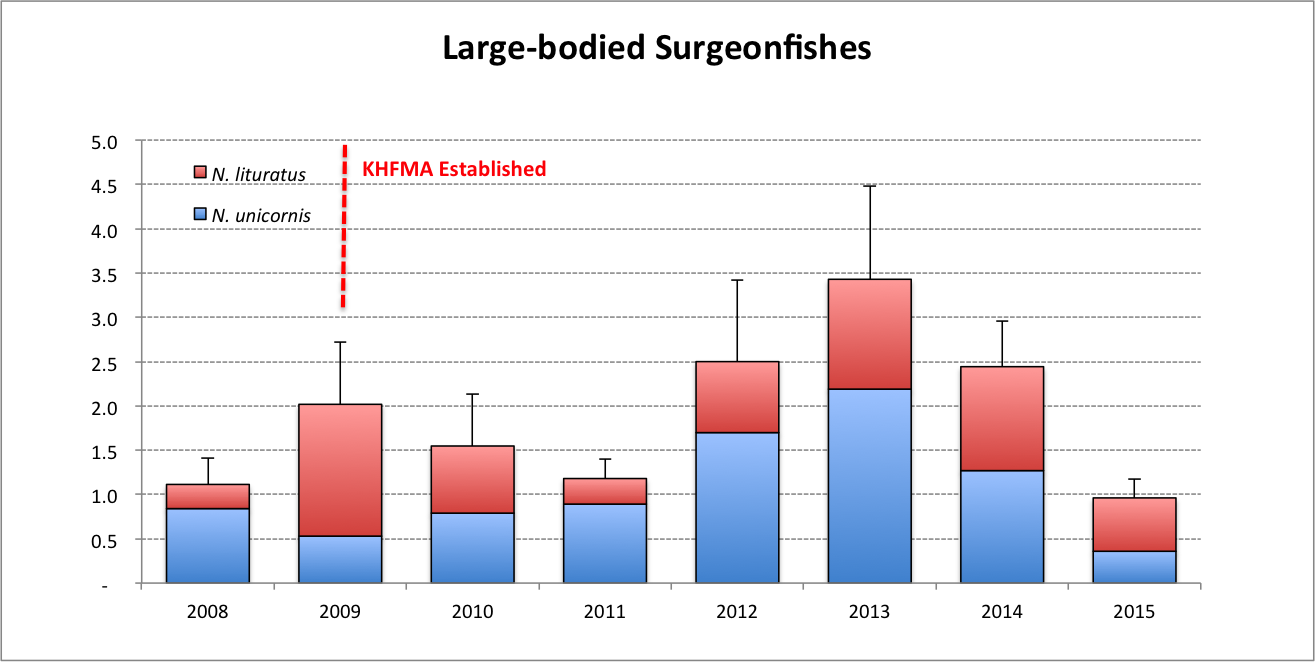 |
| --- | --- |
| 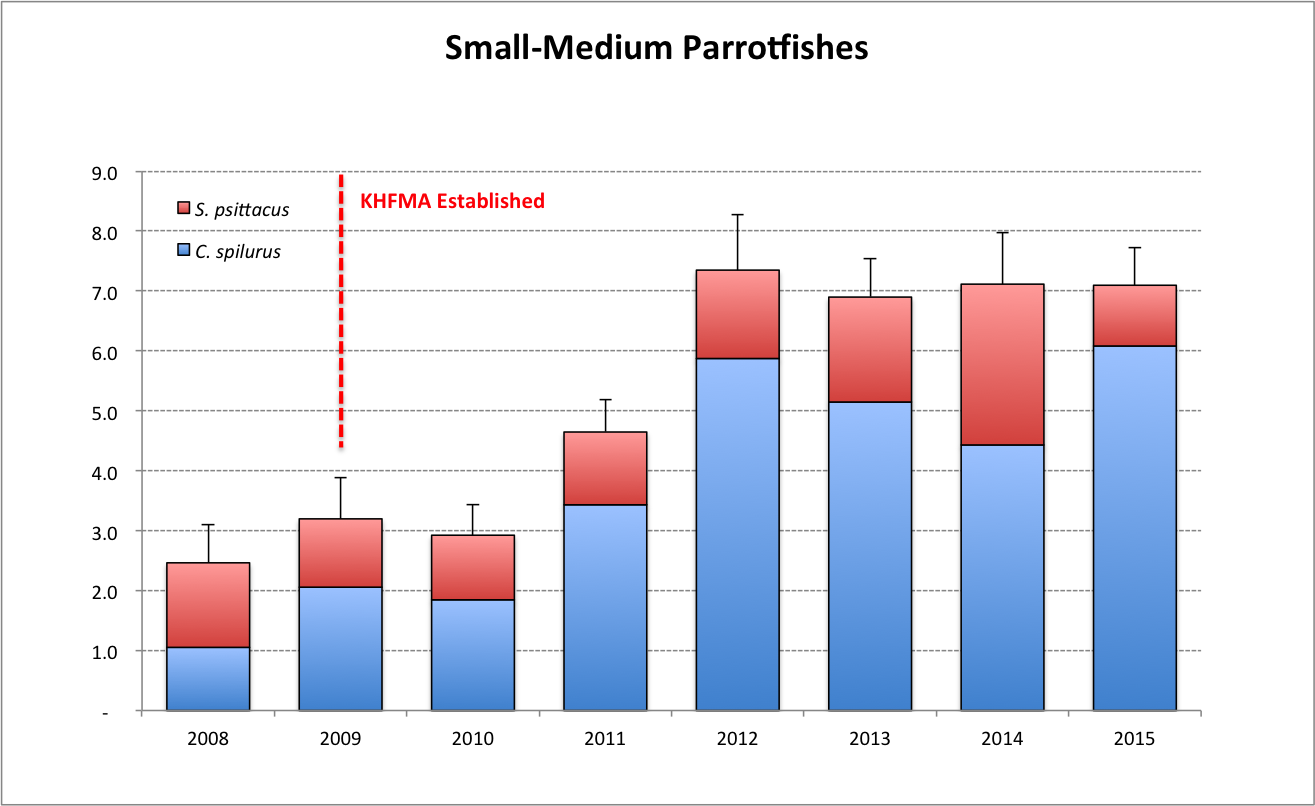 | 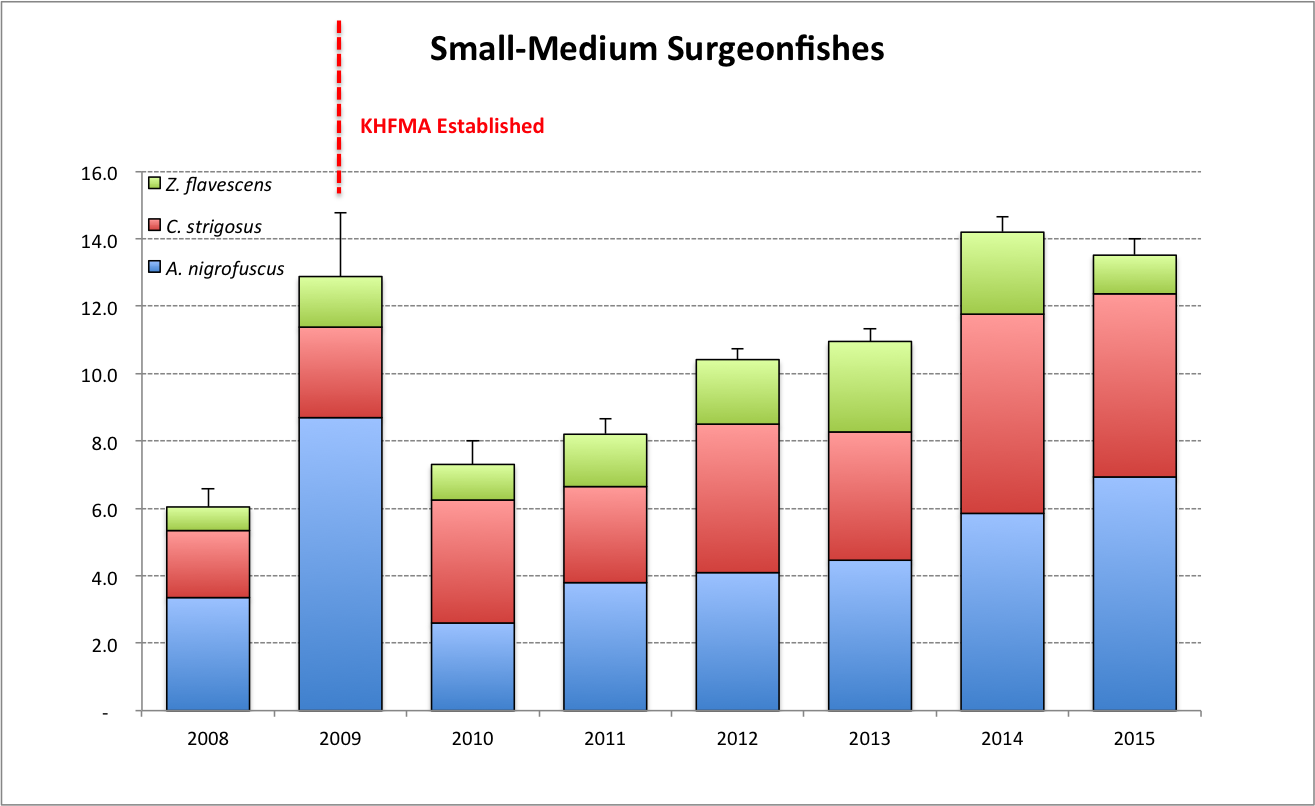 |
