# Supplementary figures and images for: Responses of Herbivorous Fishes and Benthos to 6 Years of Protection at the Kahekili Herbivore Fisheries Management Area, Maui
Source: PLoS One. 2016 Jul 27;11(7):e0159100. doi: 10.1371/journal.pone.0159100 (PMC4963024; doi:10.1371/journal.pone.0159100)

**S2 Fig. Abundance of sea urchins at KHFMA.** Data shown are annual mean and standard error.


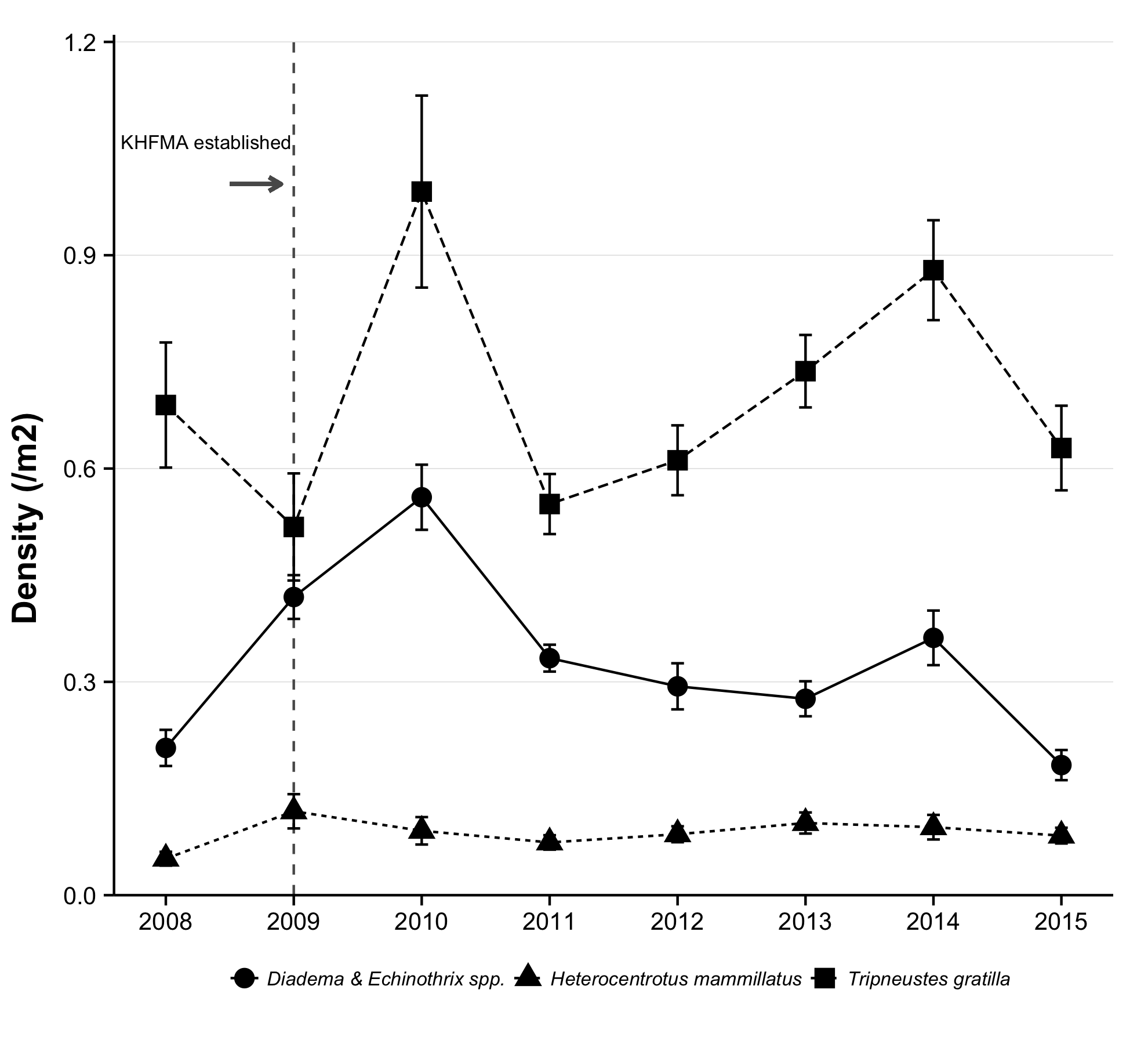

Supplement: S2 Fig — Data shown are annual mean and standard error. (DOCX) [file pone.0159100.s002.docx]
