# Supplementary material for: Responses of Herbivorous Fishes and Benthos to 6 Years of Protection at the Kahekili Herbivore Fisheries Management Area, Maui
Source: PLoS One. 2016 Jul 27;11(7):e0159100. doi: 10.1371/journal.pone.0159100 (PMC4963024; doi:10.1371/journal.pone.0159100)
Supplement: S1 Table — (DOCX) [file pone.0159100.s003.docx]

**S1 Table. Sampling Rounds. Including survey dates and # Transects.**

| **Round** | **Dates** | **# Transects** |
| --- | --- | --- |
| 1 | January 22-25 2008 | 89 |
| 2 | August 18-21 2008 | 66 |
| 3 | September 1-14 2009 | 97 |
| 4 | September 13-16 2010 | 90 |
| 5 | February 28-March 3rd 2011 | 104 |
| 6 | September 26-29 2011 | 106 |
| 7 | April 23-26 2012 | 101 |
| 8 | September 24-27 2012 | 93 |
| 9 | April 22-25 2013 | 99 |
| 10 | September 16-19 2013 | 100 |
| 11 | March 31-April 30 2014 | 72 |
| 12 | September 22-25 2014 | 88 |
| 13 | April 7-9 2015 | 62 |
| 14 | September 22-24 2015 | 74 |
