# Supplementary material for: Responses of Herbivorous Fishes and Benthos to 6 Years of Protection at the Kahekili Herbivore Fisheries Management Area, Maui
Source: PLoS One. 2016 Jul 27;11(7):e0159100. doi: 10.1371/journal.pone.0159100 (PMC4963024; doi:10.1371/journal.pone.0159100)
Supplement: S2 Table — 95% quantile range (95%QR) not overlapping zero indicates significant change at alpha = 0.05, and are shown in bold. Kahekili HFMA data are shown for all fishes recorded during Kahekili surveys, and for the subset of fishes recorded at comparable sites (i.e. fishes > 15 cm TL, and excluding two common small-bodied surgeonfishes, A. nigrofuscus and Z. flavescens. (DOCX) [file pone.0159100.s004.docx]

**S2 Table**. **Mean and SE of parrotfish and surgeonfish biomass before closure (2008-9) and in 2 most recent year (2014-15) at KHFMA and at comparative locations around Maui.** 95% quantile range (95%QR) not overlapping zero indicates significant change at alpha=0.05, and are shown in bold. Kahekili HFMA data are shown for all fishes recorded during Kahekili surveys, and for the subset of fishes recorded at comparable sites (i.e. fishes > 15 cm TL, and excluding two common small-bodied surgeonfishes, *A. nigrofuscus* and *Z. flavescens*.

|  | Parrotfishes | | 95% QR of | Surgeonfishes | | 95% QR of |
| --- | --- | --- | --- | --- | --- | --- |
| Location | 2008-9 | 2014-15 | change | 2008-9 | 2014-15 | change |
| all data |  |  |  |  |  |  |
| KHFMA | 3.4 ± 0.5 | 8.0 ± 0.6 | **+98%, +181%** | 15.6 ± 1.7 | 20.0 ± 1.1 | **+3%, +52%** |
| > 15 cm TL only |  | | |  | | |
| KHFMA | 2.7 ± 0.5 | 7.0 ± 0.5 | **+109%, + 204%** | 6.4 ± 1.0 | 5.9 ± 1.1 | -46%, +31% |
| Ahihi-Kinau NARS | 6.3 ± 1.2 | 4.6 ± 0.7 | **-**62%, +17% | 5.2 ± 0.8 | 5.2 ± 0.7 | -34%, +43% |
| Honolua MLCD | 4.4 ± 0.5 | 2.2 ± 0.3 | **-76%, -25%** | 7.1 ± 1.3 | 9.0 ± 0.8 | **-**12%, +65% |
| Kapalua Bay | 0.5 ± 0.3 | 0.9 ± 0.3 | **-**57%, +230% | 5.1 ± 1.0 | 7.5 ± 1.3 | -24%, +115% |
| La Perouse | 2.9 ± 0.8 | 1.6 ± 0.3 | **-**96%, +6% | 7.6 ± 1.9 | 12.9 ± 1.5 | **+6%, +123%** |
| Makena/Keawekapu | 3.0 ± 0.5 | 2.5 ± 0.2 | **-**52%, +25% | 7.5 ± 1.6 | 8.0 ± 1.3 | -53%, +71% |
| Molokini MLCD | 2.5 ± 0.3 | 3.1 ± 0.2 | **+2%, +55%** | 8.9 ± 1.2 | 12.5 ± 1.1 | **+6%, +83%** |
