# Supplementary material for: Responses of Herbivorous Fishes and Benthos to 6 Years of Protection at the Kahekili Herbivore Fisheries Management Area, Maui
Source: PLoS One. 2016 Jul 27;11(7):e0159100. doi: 10.1371/journal.pone.0159100 (PMC4963024; doi:10.1371/journal.pone.0159100)
Supplement: S3 Table — 95% quantile range (95%QR) not overlapping zero indicates significant change at alpha = 0.05, and are shown in bold. (DOCX) [file pone.0159100.s005.docx]

**S3 Table. Mean and SE of cover of hard coral and crustose coralline algae (CCA) before closure (2008-9) and in 2 most recent year (2014-15) at KHFMA and at comparative locations around Maui.** 95% quantile range (95%QR) not overlapping zero indicates significant change at alpha=0.05, and are shown in bold.

|  | Hard Coral % | | 95% QR of | CCA % | | 95% QR of |
| --- | --- | --- | --- | --- | --- | --- |
| Location | BEFORE | AFTER | change | BEFORE | AFTER | change |
| KHFMA | 38.0 ± 1.0 | 34.7 ± 0.6 | **-15%, -3%** | 2.5 ± 0.3 | 13.8 ± 0.6 | **+395%, +498%** |
| Honolua | 7.9 ± 0.6 | 10.1 ± 0.7 | **+4%, +52%** | 7.6 ± 1.0 | 5.2 ± 0.7 | -58%, +0% |
| Kanahena Pt. | 6.8 ± 0.6 | 19.1 ± 0.6 | **+157%, +204%** | 17.0 ± 1.8 | 12.8 ± 0.8 | **-47%, -3%** |
| Maalaea | 7.9 ± 1.0 | 9.4 ± 1.0 | -17%, +55% | 2.4 ± 0.4 | 3.9 ± 0.2 | **+26%, +97%** |
| Mahinahina | 31.0 ± 2.2 | 25.3 ± 1.5 | -29%, +3% | 5.3 ± 0.8 | 3.9 ± 0.5 | -60%, +6% |
| Molokini | 76.3 ± 1.1 | 72.9 ± 1.2 | **-9%, -0%** | 2.6 ± 0.4 | 5.0 ± 0.4 | **+51%, +136%** |
| Olowalu | 37.0 ± 1.4 | 33.4 ± 1.6 | -21%, +2% | 1.9 ± 0.2 | 4.0 ± 0.5 | **+54%, +166%** |
| Papaula Pt. | 16.3 ± 0.9 | 16.9 ± 1.0 | -12%, +19% | 14.4 ± 1.0 | 2.4 ± 0.2 | **-96%, -69%** |
| Puamana | 9.1 ± 0.9 | 7.2 ± 0.8 | -45%, +4% | 1.3 ± 0.2 | 2.0 ± 0.3 | **+6%, +107%** |
