# Supplementary material for: Responses of Herbivorous Fishes and Benthos to 6 Years of Protection at the Kahekili Herbivore Fisheries Management Area, Maui
Source: PLoS One. 2016 Jul 27;11(7):e0159100. doi: 10.1371/journal.pone.0159100 (PMC4963024; doi:10.1371/journal.pone.0159100)
Supplement: S4 Table — (DOCX) [file pone.0159100.s006.docx]

**S4 Table. Proportion of photoquadrats with >10%, >20%, >30% CCA over time**

| CCA | Proportion of photoquadrats (%) | | | | | | |
| --- | --- | --- | --- | --- | --- | --- | --- |
|  | 2008/9 | 2010 | 2011 | 2012 | 2013 | 2014 | 2015 |
| >10% | 8 | 13 | 26 | 26 | 29 | 41 | 46 |
| >20% | 4 | 3 | 12 | 12 | 13 | 21 | 28 |
| >30% | 2 | 1 | 8 | 7 | 8 | 14 | 20 |
| >40% | 1 | 0 | 4 | 3 | 4 | 7 | 10 |
| >50% | 0 | 0 | 2 | 2 | 2 | 5 | 6 |
